# Radiotherapy plus temozolomide in elderly patients with glioblastoma: a “real-life” report

**DOI:** 10.1186/s13014-017-0929-2

**Published:** 2017-12-06

**Authors:** J. Biau, E. Chautard, E. De Schlichting, G. Dupic, B. Pereira, A. Fogli, M. Müller-Barthélémy, P. Dalloz, T. Khalil, A. F. Dillies, X. Durando, C. Godfraind, P. Verrelle

**Affiliations:** 1 Radiotherapy Department, Université Clermont Auvergne, Centre Jean Perrin, 63011 Clermont-Ferrand, France; 2 Université Clermont Auvergne, INSERM, U1240 IMoST, F-63000 Clermont Ferrand, France; 3 Neurosurgery Department, Clermont-Ferrand Hospital, 63003 Clermont-Ferrand, France; 4 Biostatistics Department, DRCI, Clermont-Ferrand Hospital, 63003 Clermont-Ferrand, France; 5 Université Clermont Auvergne, CNRS UMR 6293, INSERM U1103, GReD Laboratory, 63000 Clermont-Ferrand, France; 6 Oncology Department, Université Clermont Auvergne, Centre Jean Perrin, 63011 Clermont-Ferrand, France; 7 Anatomopathology Department, Clermont-Ferrand Hospital, 63003 Clermont-Ferrand, France; 8 Radiation Oncology Department, Institut Curie, 75248 Paris, France

**Keywords:** Elderly, Glioblatoma, Temozolomide, Hypofractionated radiotherapy

## Abstract

**Background:**

The optimization of the management for elderly glioblastoma patients is crucial given the demographics of aging in many countries. We report the outcomes for a “real-life” patient cohort (i.e. unselected) comprising consecutive glioblastoma patients aged 70 years or more, treated with different radiotherapy +/− temozolomide regimens.

**Methods:**

From 2003 to 2016, 104 patients ≥ 70 years of age, consecutively treated by radiotherapy for glioblastoma, were included in this study. All patients were diagnosed with IDH-wild type glioblastoma according to pathological criteria.

**Results:**

Our patient cohort comprised 51 female patients (49%) and 53 male. The median cohort age was 75 years (70–88), and the median Karnofsky performance status (KPS) was 70 (30–100). Five (5%) patients underwent macroscopic complete resection, 9 (9%) had partial resection, and 90 (86%), a stereotactic biopsy. The *MGMT* promoter was methylated in 33/73 cases (45%). Fifty-two (50%), 38 (36%), and 14 (14%) patients were categorized with RPA scores of III, IV, and I-II. Thirty-three (32%) patients received normofractionated radiotherapy (60 Gy, 30 sessions) with temozolomide (Stupp), 37 (35%) received hypofractionated radiotherapy (median dose 40 Gy, 15 sessions) with temozolomide (HFRT + TMZ), and 34 (33%) HFRT alone. Patients receiving only HFRT were significantly older, with lower KPSs. The median overall survival (OS; all patients) was 5.2 months. OS rates at 12, 18, and 24 months, were 19%, 12%, and 5%, respectively, with no statistical differences between patients receiving Stupp or HFRT + TMZ (*P* = 0.22). In contrast, patients receiving HFRT alone manifested a significantly shorter survival time (3.9 months vs. 5.9 months, *P* = 0.018). In multivariate analyses, the prognostic factors for OS were: i) the type of surgery (HR: 0.47 [0.26–0.86], *P* = 0.014), ii) RPA class (HR: 2.15 [1.17–3.95], P = 0.014), and iii) temozolomide use irrespective of radiotherapy schedule (HR: 0.54 [0.33–0.88], *P* < 0.02). *MGMT* promoter methylation was neither a prognostic nor a predictive factor.

**Conclusions:**

These outcomes agree with the literature in terms of optimal surgery and the use of HFRT as a standard treatment for elderly GBM patients. Our study emphasizes the potential benefit of using temozolomide with radiotherapy in a real-life cohort of elderly GBM patients, irrespective of their *MGMT* status.

## Background

Glioblastoma (GBM) is the most common adult primary brain tumor [1]. According to the World Health Organization (WHO) classification, now updated to include molecular features such as Isocitrate DeHydrogenase (IDH) mutation, GBM is the most malignant astrocytic tumor (Grade IV) [2]. The incidence of GBM increases with age, with approximately half of all GBM patients aged over 65 years, and 20% older than 75 years [3].

Improved diagnoses due to the standard use of non-invasive techniques (e.g. MRI), combined with stereotactic biopsy, explain, in part, the increasing incidence of GBM diagnosed in elderly patients [4]. Optimized management for this patient group is fast becoming a significant consideration, especially given the current demographic scenario of increasingly aged populations in Western nations. However, an obstacle in analyzing patient care for this cohort is its heterogeneity, which can complicate the identification of patients who could genuinely benefit from standard treatment.

For elderly patients with GBM, their median survival is drastically reduced compared to their younger counterparts [5–14]. This low median survival could be explained, in part, by an unfavorable tumor biology, a trend towards less aggressive treatment, treatment toxicity, performance status, and comorbidities [15, 16]. Because of their poor prognoses, treatment efficiency and quality of life are of major interest in the management of these patients.

Radiotherapy results in a modest improvement in survival compared to supportive care alone in GBM patients aged 70 years or more with a good performance status, and does not reduce the quality of life or cognition [5]. Due to their poor prognosis, the time taken for normofractionated radiotherapy could constitute a third of the life expectancy for this patient group. Further, Roa et al. [6] showed that normofractionated radiotherapy (60 Gy, 30 sessions) failed to improve survival compared to hypofractionated radiotherapy (HFRT; 40 Gy, 15 sessions) in GBM patients aged 60 years or more. Further, no increased toxicity was noted with HFRT [9]. Other treatments such as chemotherapy alone, gave favorable results compared to radiotherapy, notably for elderly patients with *MGMT* methylation [7, 8]. The use of temozolomide (TMZ) treatment alongside radiotherapy and adjuvant TMZ, which is the standard treatment for younger patients [17, 18], has been debated at length for elderly GBM patients [10, 13, 19, 20]. The recent results of a large phase 3 trial appeared to emphasize the role of TMZ with HFRT, especially for patients aged over 70 [14].

Although radiotherapy +/− TMZ has shown potential benefits in the management of elderly GBM patients in randomized trials [5–8, 14], the ability to evaluate its benefit in routine clinical practice may be limited by the selective patient populations that participate in trials (reflecting trial eligibility criteria), as well as patient sample sizes, and reporting bias [20–22]. Thus, the aim of our study was to report the outcomes of elderly GBM IDH-wild-type patients aged 70 years or more, treated consecutively with different radiotherapy +/− TMZ regimens in a single institution. Critically, this study reports data for a real-life, unselected, elderly patient population.

## Methods

### Patient selection

From February 2003 to June 2016, 104 patients aged 70 years or more, consecutively treated by radiotherapy for a GBM in Centre Jean Perrin, were included in this study. For all patients a surgery (stereotactic biopsy, partial or complete resection) permitted a histopathological analysis of the tumor to establish a GBM diagnosis without IDH mutation (by immunohistochemistry).

### Radiotherapy

For irradiation, all patients were immobilized with custom thermoplastic masks. Target volume and organs at risk delineation was performed by a dedicated CT-scan in the treatment position matched and fused with contrast enhanced T1-weighted and Flair MRI sequences. The gross tumor volume (GTV) was defined as the contrast enhancement area in the T1-weighted MRI sequence and/or CT-scan, including the tumor bed for patients with partial or complete resection. The clinical target volume (CTV) was defined as the addition of a geometric tridimensional one-cm margin around the GTV that was corrected to the anatomical borders [23–25]. The CTV also included the hyper intensity in the Flair MRI sequence. The planning target volume (PTV) was defined as the addition of a geometric tridimensional 4-mm margin around the CTV. Radiotherapy was administrated according to a Stupp normofractionated regimen of 60 Gy in 30 sessions (EORTC (26981–22,981)/NCIC CTG (CE.3)) [17], or according to a hypofractionated regimen (HFRT, 30–45 Gy in 10 to 15 sessions). For patients receiving concomitant chemotherapy, TMZ was administrated at a dose of 75 mg/m^2^ daily (7 days a week, from the first to last radiotherapy session). For patients receiving adjuvant chemotherapy, the TMZ dose ranged from 150 to 200 mg/m^2^ per day, given on five consecutive days per month.

### *MGMT* methylation analysis

For 73/104 (70%) tumor DNA samples, *MGMT* methylation was analyzed using the PyroMark MGMT kit (Qiagen). Chemically methylated and unmethylated human genomic DNA controls (EpiTect PCR Control DNA Set, Qiagen) were included in each batch. In brief, 40 ng of tumor DNAs were extracted from paraffin-embedded tissue blocks using the FFPE DNA Extraction Kit (Zymo Research, Orange, CA). DNAs were then bisulfite-modified using the EZ DNA methylation kit (Zymo Research, Orange, CA) according to the manufacturer’s recommendations. The CpG pyrosequencing methylation assay using the Qiagen kit was performed on a PSQ 96 MA system (Qiagen) according to the manufacturer’s protocol. The PyroMark MGMT kit quantifies the level of methylation at five individual CpG sites within exon 1 of *MGMT* using the Pyromark CpG software (Qiagen). The *MGMT* promoter was defined as unmethylated when the mean methylation of the five CpG sites was <8%, and methylated when this value was ≥ 8% [26].

### Statistical analyses

Statistical analyses were performed using R v2.15.1 (http://www.cran.r-project.org). Tests were two-sided, with a type I error set at α = 0.05. Baseline characteristics are presented as median values [interquartile range] for each independent group for continuous data, and as the number of patients and the associated percentages for categorical parameters. Quantitative variables (age, KPS) were categorized according to clinical relevance. Categorical variables were compared between groups (Stupp, HFRT + TMZ, HFRT) using the Chi-squared or Fisher’s exact tests, followed, as appropriate (*P*-value < 0.05), by post-hoc tests for multiple comparisons (Marascuilo approach). For quantitative parameters, ANOVA and Kruskal-Wallis (KW) tests were performed according to ANOVA assumptions (normality verified by the Shapiro–Wilk test and homoscedasticity by the Bartlett test). If the P-value was < 0.05, a post-hoc test was considered: Tukey-Kramer post ANOVA, or Dunn after KW. OS was defined as the interval from surgery to death, regardless of the cause of death. OS curves and estimates were constructed using the Kaplan–Meier method. To test the prognostic value of the patients’ characteristics in the univariate context, OS curves were compared between groups using Cox proportional hazards regression. Finally, to evaluate the impact of treatment (Stupp, HFRT + TMZ, HFRT) on OS, multivariate analyses were performed using Cox proportional hazards regression to take into account adjustment for possible confounding factors as determined by univariate analysis and clinical relevance. Two multivariate models were constructed: model 1 with KPS, age, surgery (complete or partial resection vs biopsy only) and gender, and model 2 with RPA class and gender. Results are expressed as hazard ratios (HRs) with 95% confidence intervals (95%-CIs).

## Results

### Patient characteristics and treatment

The characteristics of the 104 IDH-wild-type GBM patients are summarized in Table 1. The median age was 75 years (range, 70–88 years), with 51 female patients (49%), and 53 male (51%). The median Karnofsky performance status (KPS) was 70 (range, 30–100). According to Scott et al., the patients were then classified by their RPA (recursive partitioning analysis) class, specific for elderly patients [27]. RPA classes I and II were pooled to present data. Fifty-two patients (50%), 38 (36%), and 14 (14%), were categorized as RPA class III, IV, and I-II, respectively. A macroscopic complete resection was realized for 5 patients (5%), 9 patients (9%) underwent partial resection, and 90 patients (86%) underwent stereotactic biopsy. *MGMT* promoter methylation was assessed in 73/104 cases (70%), with methylation confirmed in 33 cases (45%); the remaining 40 cases were unmethylated (55%).

**Table 1:** Patient characteristics

		Treatment			
	Total	Stupp	HFRT + TMZ	HFRT	*p*
Number of patients	104	33 (32%)	37 (35%)	34 (33%)	
Gender					0.65
*Male*	53 (51%)	19 (58%)	18 (49%)	16 (47%)	
*Female*	51 (49%)	14 (42%)	19 (51%)	18 (53%)	
Age					<0.01*
*Median (years)*	75 [70–88]	73 [70–81]	75 [70–80]	79 [70–88]	
*< 75.5 years*	55 (53%)	24 (73%)	20 (54%)	11 (32%)	
≥ *75.5 years*	49 (47%)	9 (27%)	17 (46%)	23 (68%)	
KPS					<0.01*
Median	70 [30–100]	80 [50–100]	70 [30–100]	60 [40–90]	
*KPS < 70*	40 (38%)	5 (15%)	13 (35%)	22 (65%)	
*KPS* ≥ *70*	64 (62%)	28 (85%)	24 (65%)	12 (35%)	
Type of suregery					0.09
*Resection (complete/ partial)*	5 / 9 (5% / 9%)	2 / 6 (6% / 18%)	1 / 2 (3% / 5%)	2 / 1 (6% / 3%)	
*Biopsy*	90 (86%)	25 (76%)	34 (92%)	31 (91%)	
MGMT status					0.58
*Methylated*	33 (45%)	11 (41%)	12 (43%)	10 (56%)	
*Unmethylated*	40 (55%)	16 (59%)	16 (57%)	8 (44%)	
*Unknown*	31	6	9	16	
RPA Class					<0.001*
*I-II*	14 (14%)	8 (24%)	3 (8%)	3 (9%)	
*III*	52 (50%)	20 (61%)	22 (59%)	10 (29%)	
*IV*	38 (36%)	5 (15%)	12 (32%)	21 (62%)	
Adjuvant Temozolomide	28 (27%)	18 (55%)	10 (10%)	0 (0%)	<0.0001*
Treatment at recurrence	12 (12%)	7 (21%)	3 (8%)	2 (6%)	0.1

*Abbreviations: HFRT* Hypofractionated Radiotherapy, *TMZ* Temozolomide, *KPS* Karnofsky Performance. *: significant difference between HFRT and Stupp/HFRT + TMZ. No statistical difference was found between Stupp and HFRT + TMZ regarding all characteristics

All of the patients received radiotherapy. Thirty-three patients (32%) adhered to a normofractionated schedule (60 Gy, 30 sessions) with TMZ used according to the Stupp protocol. Thirty-seven patients (35%) received HFRT with TMZ (HFRT + TMZ), and 34 patients (33%) received HFRT alone (HFRT). The HFRT regimen ranged from 30 to 45 Gy in 10 to 15 sessions (median 40 Gy in 15 fractions). Adjuvant TMZ was used for 28 patients (27%) with a median number of cycles of 4 (range, 1–12). Patients receiving the Stupp protocol or HFRT + TMZ showed no statistical differences in terms of age, KPS, type of surgery, *MGMT* status, RPA class, and adjuvant TMZ treatment (Table 1). In contrast, patients receiving HFRT alone were significantly older, with a lower KPS (median of 60), and had received less adjuvant treatment.

### Overall survival and prognostic factors

**Fig. 1:**
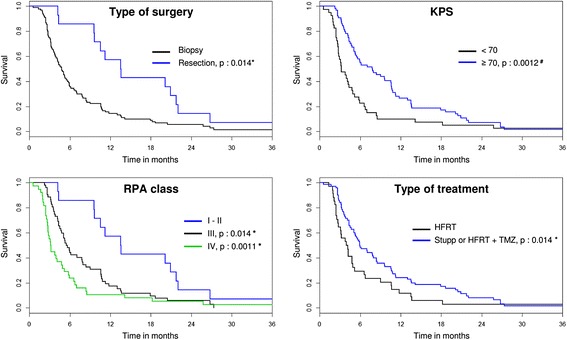
Prognostic factors for elderly GBM patients. Kaplan–Meier survival curves for patients’ classified according to type of surgery, Karnofsky Performance Status (KPS), RPA class, and type of treatment. The *P*-values of the prognostic factors are indicated for univariate (#) or multivariate (*) analyses. HFRT: Hypofractionated radiotherapy, TMZ: Temozolomide

For the entire patient population, the median overall survival (OS) was 5.2 months (Table 2). The OS rates at 12, 18, and 24 months were 19%, 12%, and 5%, respectively. No statistically significant differences were found between the Stupp protocol and HFRT + TMZ (*P* = 0.22). In contrast, patients receiving HFRT alone manifested a significantly shorter survival time (3.9 months vs. 5.9 months, *P* < 0.05) (Fig. 1).

**Table 2:** Survival and prognostic factors

	Median survival (months)	12 months survival (%)	Univariate analysis		Multivariate analysis	
			HR [95%CI]	*p*	HR [95%CI]	*p*
Age						
*< 75.5 years*	5.6	18.6				
≥ *75.5 years*	5.1	17.8	1.03 [0.69–1.53]	0.88	0.76 [0.49–1.20]	0.25 ^# $^
KPS						
*< 70*	3.2	7.5				
≥ *70*	7.8	25	0.52 [0.34–0.77]	< 0.01	0.70 [0.45–1.09]	0.11 ^#^
Gender						
*Female*	4.5	15.7				
*Male*	6.1	20.7	0.70 [0.47–1.05]	0.08	0.71 [0.47–1.08]	0.10 ^# $^
MGMT status						
*Methylated*	5.9	12.1				
*Unmethylated*	4.8	17.5	1.14 [0.72–1.82]	0.57		
Type of surgery						
*Biopsy*	4.8	50				
*Resection*	13.5	13	0.43 [0.24–0.77]	< 0.05	0.47 [0.26–0.86]	< 0.05 ^#^
RPA class						
*I-II*	13.5	50				
*III*	5.5	15.4	1.95 [1.07–3.57]	< 0.05 *	2.15 [1.17–3.95]	< 0.05 ^$^ *
*IV*	3.1	7.9	3.08 [1.65–5.76]	< 0.001 *	2.87 [1.53–5.41]	< 0.01 ^$^ *
Type of treatment						
*HFRT + TMZ*	5.5	18.9				
*Stupp*	9.6	24.2	0.74 [0.46–1.20]	0.22^¤^		
*HFRT*	3.9	8.8				
*HFRT + TMZ or Stupp*	5.9	22.9	0.6 [0.40–0.92]	< 0.05^§^	0.54 [0.33–0.88]	< 0.05 ^# $ §^

*Abbreviations: HR* hazard ratio, *HFRT* Hypofractionated radiotherapy, *TMZ* Temozolomide, *KPS* Karnofsky Performance Status. #: Multivariate analysis model 1 adjusted for age, gender, KPS and type of surgery, $: Multivariate analysis model 2 adjusted for gender, and RPA class.* compared with RPA class I-II. ¤ compared with HFRT+TMZ; §: compared with HFRT

In the univariate analyses (Table 2), the prognostic factors of OS were: KPS (< 70 vs. ≥ 70; median survival of 3.2 vs. 7.8 months; *P* < 0.01), the type of surgery (biopsy vs. partial or complete resection; median survival of 4.8 vs. 13.5 months; *P* < 0.05), RPA class (median survival of 13.5 months for class I-II vs. 5.5 months for class III (*P* < 0.05) or 3.1 months for class IV (*P* < 0.001)), and the use of TMZ irrespective of radiotherapy schedule (median survival of 3.9 months vs. 5.9 months, P < 0.05). Age and gender were not statistically significant prognostic factors. *MGMT* status has no impact on overall survival on the entire cohort (prognostic factor) and on the TMZ-treated population (predictive factor).

Two models were constructed for the multivariable analysis to evaluate the use of TMZ, regardless of radiotherapy schedule: model 1 adjusted for age, gender, KPS, and type of surgery (complete or partial resection vs biopsy only); and model 2 adjusted for gender, and RPA class. In both multivariate models, the use of TMZ irrespective of radiotherapy schedule was a prognostic factor of OS (HR: 0.54 [0.33–0.88], *P* < 0.05). The type of surgery (HR: 0.47 [0.26–0.86], *P* < 0.05) and RPA class (HR: 2.15 [1.17–3.95], P < 0.05 for RPA class III; HR: 2.87 [1.53–5.41], *P* < 0.01 for RPA class IV) remained prognostic factors of OS in their respective multivariate models.

## Discussion

In our study we selected GBM IDH-wild-type patients aged 70 years or more, according to the new WHO classification [2], who were treated consecutively with radiotherapy. The median overall survival in our study of 5.2 months agrees with other studies of elderly GBM patients [5–10, 12]. Roa et al. [6], in their assessment of the impact of radiotherapy regimens, found a similar median survival when using normofractionated radiotherapy (5.1 months) and HFRT (5.6 months). Three recent population-based studies of 1652, 5252, and 5575 elderly GBM patients reported median survivals of between 5 and 7.4 months, depending on the type of treatment [10, 12, 20]. As previously reported, our study shows that a KPS score < 70 is associated with a poorer prognosis (median survival: 3.2 months), underlining the need to consider an active therapy on a case-by-case basis.

Our study suggests improved survival with partial or complete surgical resection compared to biopsy, as reported by others in retrospective studies in elderly GBM patients [28, 29]. Caution should be used when interpreting these data as only 14 patients had complete or partial resection vs 90 biopsy only. Furthermore, biopsy is ordinarily offered to patients unsuitable for partial or complete surgery. Only one prospective study by Vuorinen et al. evaluated the impact of surgery in elderly patients [30].With a reduced cohort, they reported an improvement of median survival after surgical resection (5.6 months) compared to biopsy (2.8 months). A prospective randomized multicentric study (CSA NCT02892708) is ongoing in operable patients over 70 years of age, and will compare surgical resection and biopsy.

Another interesting aspect of these data concerns the type of radiotherapy fractionation. We found no survival differences when comparing patients treated with HFRT + TMZ vs the Stupp regimen. These results agree with data reported by Roa et al. in a randomized trial evaluating HFRT and normofractionated radiotherapy [6]. Thus, according to the guidelines, the HFRT regimen is more than a therapeutic alternative to the normofractionated regimen for elderly patients [19]. However, this randomized trial was conducted before the era of TMZ. Recently, Lombardi et al. [11] published a large multicenter retrospective study comparing short (HFRT; 40 Gy, 15 sessions) or standard-course (60 Gy, 30 sessions) irradiation plus concomitant TMZ in elderly (≥ 60 years) GBM patients (Table 3). Patients receiving HFRT were older (*p* = 0.07). This study suggested that standard-course irradiation + TMZ might be more effective than HFRT + TMZ. The median overall survival in this study was remarkably high with 17.3 months; with even 19.4 months for patients treated with the Stupp regimen (4.8 months more than in the original study by Stupp et al. [17]). This could maybe be explained by the relatively strict inclusion criteria in this retrospective study with no patients with biopsy only (70–80% of patients with complete resections) and 82% of patients ECOG 0–1, not reflecting “real life” data as in our study. Furthermore, average age in this study was 71 years while our study only included patients 70 years or more, with a median age of 75 years. However, this study brings interesting data concerning “moderate” elderly patients, in excellent general status, who had extensive surgery and for which a Stupp regimen might considered over HFRT + TMZ. Minniti et al. [31] also compared the outcomes of elderly (≥ 65 years) GBM patients receiving either short (HFRT; 40 Gy, 15 sessions) or standard-course (60 Gy, 30 sessions) irradiation plus concomitant TMZ (Table 3). To limit the potential bias of such a retrospective study, they designed a propensity-matched analysis. Patients given standard-course irradiation + TMZ were more likely to be younger (median age of 68 years vs 71) and to undergo total/subtotal resection. After propensity score-matching analysis, they found no differences in overall survival or progression-free survival between both groups.

**Table 3:** Major studies regarding elderly glioblastoma patients

Major interest	Reference	Year	Design	Number of patients	Age	KPS	Treatment	Median survival in months (*p*)	Notes
Surgery	Vuorien et al.[30]	2003	Prospective	23	≥ 65	> 60	Biopsy + RT	2.8	Longer survival after resection while time to neurological deterioration did not differ.
							Resection + RT	5.7 (*< 0.05*)	
Radio-therapy	Roa et al.[6]	2004	Prospective	100	≥ 60	≥ 50	RT	5.1	Half overall treatment time for HFRT with no difference in survival.
							HFRT	5.6 (*ns*)	
	Keime-Guibert et al. [5]	2010	Prospective	81	≥ 70	≥ 70	Supportive care	3.9	No negative effect of RT on quality of life.
							RT	6.7 (< *0.01*)	
Radio-therapy + TMZ	Minniti et al. [34]	2009	Prospective	43	≥ 70	≥ 60	HFRT + TMZ	9.3	Grade 3–4 hematologic toxicity occurred in 28% of patients. no negative effect on quality of life.
	Minniti et al. [33]	2012	Prospective	71	>70	> 60	HFRT + TMZ and TMZ	12.4	Grade 3–4 hematologic toxicity occurred in15% of patients.
	Minniti et al. [31]	2015	Retrospecitve	127	≥ 65	≥ 60	RT + TMZ and TMZ	12	No difference in overall survival or progression free survival between standard RT and HFRT
							HFRT + TMZ and TMZ	12.5 *(ns)*	
	Lombardi et al. [11]	2015	Retrospecitve	237	≥65	ECOG PS 0–2	HFRT + TMZ	13.8	Potential advantage of standard RT over HFRT for “moderate” elderly patients with good clinical status and extensive surgery
							RT + TMZ	19.4 *(p = 0.02)*	
	Perry et al.[14]	2017	Prospective	562	≥65	ECOG PS 0–2	HFRT	7.6	The addition of TMZ (concomitant and adjuvant) to HFRT resulted in longer overall survival than HFRT alone
							HFRT + TMZ and TMZ	9.3 *(p < 0.001)*	
	Present study	2017	Retrospective	104	≥ 70	≥ 30	HFRT	3.9 (p < 0.05)*	Potential benefit of combining TMZ with RT in an unselected cohort, irrespective of MGMT promoter status.
							HFRT + TMZ	5.5	
							RT + TMZ	9.6 (ns)**	
HFRT alone	Guedes de Castro et al. [32]	2017	Prospective	61	≥ 65	≥ 50	HFRT 40Gy in 15 fractions	6.2	HFRT of 25Gy in 5 fractions seemed acceptable especially for elderly patients with a poor performance status or contraindication to chemotherapy.
							HFRT 25Gy in 5 fractions	9.1	
TMZ alone	Wick et al. [8]	2012	Prospective	371	> 65	≥ 60	Dose-dense TMZ alone	8.0	MGMT methylation is a predictive marker of TMZ alone efficacy.
							RT	9.6 (*ns*)	
	Malmström et al. [7]	2012	Prospective	291	≥ 60	OMS 0–2	TMZ alone	8	No benefit of RT over HFRT. *MGMT* methylation is a predictive marker of TMZ alone efficacy.
							HFRT	7.5	
							RT	6	
Poor perfor-mance status	Gállego Pérez-Larraya et al. [35]	2011	Prospective	70	≥ 70	<70	TMZ alone	5.8	KPS improvement in 30% of patients by 10 or more points.
	Reyes-Botero et al. [36]	2013	Prospective	66	≥ 70	<70	TMZ + Bevacizumab	5.5	Lower safety of the combination of TMZ with bevacizumab, no survival benefit

*HFRT* Hypofractionated radiotherapy, *TMZ* Temozolomide, *KPS* Karnofsky Performance Status, *MGMT* methyl-guanine methyl-transferase, *Gy* Gray. * HFRT vs HFRT or RT + TMZ ** HFRT + TMZ vs standard RT + TMZ

Recently, Guedes de Castro et al. [32] compared 2 short-course radiation therapy regimens in elderly (65 years or older) glioblastoma patients: 25 Gy in 5 fractions vs 40 Gy in 15 fractions. This trial was conducted before the results of Perry’s trial [14] showing the superiority of combining TMZ with HFRT over HFRT alone. The short-course HFRT (25 Gy in 5 fractions) results were not statistically significantly different from the results of commonly used HFRT (40Gy in 15 fractions) (median overall survival of 6.8 months vs 6.2 months; *p* = 0.936). The authors concluded that a short-course HFRT regimen of 25 Gy in 5 fractions was an acceptable treatment option for patients aged ≥ 65 years, mainly those with a poor performance status or contraindication to chemotherapy.

Several studies have shown that *MGMT* methylation appears to be to an important prognostic factor in the management of elderly GBM patients, and a predictive factor of response to radiotherapy + TMZ [11, 13, 14]. In our study, the proportion of patients with methylated *MGMT* is similar to the published data. However, we failed to find that *MGMT* methylation was either a prognostic or predictive factor. This discrepancy could, in part, be explained by our smaller cohort size and the high rate of biopsy. *MGMT* methylation was described as predictive of TMZ treatment response in elderly patients with a good performance status in two randomized phase III trials comparing TMZ alone with various radiotherapy regimens (Nordic trial [7] and the German trial NOA-08 [8]). However, none of our patients underwent this type of treatment.

We also report that the use of TMZ, irrespective of radiotherapy regimen, and irrespective of the MGMT promoter status, was a positive prognostic factor in multivariate analysis. A phase II clinical trial published by Minniti et al. tested HFRT + TMZ (40 Gy, 15 fractions) and adjuvant TMZ in patients aged over 70 years and with a KPS score > 60 [33]. They reported a 22% rate of grade III-IV toxicities linked to TMZ uptake, the majority (15%) constituting hematologic toxicity (4% in adjuvant TMZ). The median survival was 12.4 months. OS rates at 12 and 24 months were 58% and 20%, respectively. Perry et al. published a phase III clinical trial comparing HFRT +/− TMZ in patients of 65 years of age or older [14]. The median survival time increased from 7.6 months for radiotherapy alone to 9.3 months for the combined treatment (*P* < 0.001). This increased survival was more pronounced for patients aged over 70 years with a methylated *MGMT* promoter but was also present for unmethylated MGMT promoter. In our study, the median survival of patients treated by HFRT + TMZ was 5.5 months with 1-year OS rate of 19%. However, our cohort demonstrated a higher rate of stereotactic biopsy (92%) compared to either Minniti et al. (13%) or Perry et al. (31.7%) [14, 33].

As regards to the literature reported here, and the results of our “real life” report, HFRT + TMZ appears as a major option for elderly GBM patients, regardless of MGMT promoter status, with a favorable balance between efficacy and quality of life. For patients unsuitable for TMZ but suitable for radiotherapy, HFRT alone may still have a role. For “moderate” elderly patients, with excellent general status who underwent extensive resection and thus have a better prognosis, a Stupp regimen might be considered.

## Conclusions

The outcomes reported in this study agree with the literature in terms of the use of optimal surgery as well as HFRT as a standard treatment for elderly GBM patients with KPS scores ≥ 70. Our study underscores the potential benefits to elderly GBM patients of combining TMZ with radiotherapy in an unselected cohort, irrespective of *MGMT* promoter status.

## Abbreviations

CTV: Clinical Target Volume
GBM: Glioblastoma
GTV: Gross Tumor Volume
HFRT: HypoFractionated RadioTherapy
IDH: Isocitrate DeHydrogenase
KPS: Karnofsky Performance Status
MGMT: O6-MethylGuanine-DNA-MethylTransferase
OS: Overall Survival
PTV: Planning Target Volume
RPA: Recursive Partitioning Analysis
TMZ: Temozolomide
WHO: World Health Organization
